# Virtual Reality Exposure Therapy and Patient Education for Preoperative Anxiety in Pediatrics: Randomized Controlled Trial

**DOI:** 10.2196/73392

**Published:** 2025-10-27

**Authors:** Sebastian Amaya, Sidhant Kalsotra, Sibelle Aurelie Yemele Kitio, Joseph Drew Tobias, Brittany Willer

**Affiliations:** 1 Department of Anesthesiology, School of Medicine, Yale University, 333 Cedar St, New Haven, New Haven, CT, 06510, United States, 1 6028819133; 2 Anesthesiology and Critical Care Interest Group, School of Medicine, Universidad El Bosque, Bogotá, Colombia; 3 Department of Anesthesiology and Pain Medicine, Nationwide Children's Hospital, Columbus, OH, United States; 4 Department of Anesthesiology and Pain Medicine, The Ohio State University Wexner Medical Center, Columbus, OH, United States

**Keywords:** anesthesiology, pediatrics, anxiety, virtual reality, exposure therapy

## Abstract

**Background:**

The perioperative environment is complex and may be challenging for patients and guardians to navigate. The emotional burden and stressors inherent to the perioperative process commonly result in preoperative anxiety. Many studies have demonstrated the usefulness of virtual reality (VR) in various patient populations.

**Objective:**

The aim of this study is to evaluate the impact of a VR-based preoperative education tool on anxiety levels in pediatric patients undergoing ambulatory ear, nose, and throat surgery, as well as in their guardians.

**Methods:**

We performed a single-center prospective randomized controlled trial including children 6‐12 years of age, presenting for ambulatory tonsillectomy and/or adenoidectomy, with or without bilateral ear tube insertion. The patients were randomized to receive VR instruction of the perioperative workflow or standard preoperative experience (non-VR). The primary outcome was patient and guardian preoperative anxiety, as measured by the 6-item State-Trait Anxiety Inventory.

**Results:**

The study cohort included 107 patient-guardian dyads—51 in the intervention (VR) group and 56 in the control (non-VR) group. Baseline characteristics between the study and control groups were comparable; however, patients in the control group were more likely to report feeling upset compared to the VR group. The VR intervention was associated with reduced preoperative anxiety in patients and guardians compared to the control group. Patients exposed to the VR intervention had higher odds of feeling calm (OR 4.95, 95% CI 2.32‐10.61; *P*<.001) and lower odds of feeling worried (OR 0.25, 95% CI 0.12‐0.53; *P*<.001) compared to the control group. Similarly, guardians in the VR group had higher odds of feeling calm (OR 3.55, 95% CI 1.69‐7.49; *P*=.001) and lower odds of feeling worried (OR 0.45, 95% CI 0.22‐0.93; *P*=.03) compared to the control group. Both patients and guardians exposed to VR were significantly less likely to have moderate or high levels of preoperative anxiety than the control group (patients: OR 0.15, 95% CI 0.05‐0.41, *P*<.001; guardians: OR 0.14, 95% CI 0.06‐0.38, *P*<.001).

**Conclusions:**

VR exposure may be effective in reducing pediatric and guardian anxiety. VR may be a suitable alternative to pharmacologic anxiolysis and future studies should compare the effect to premedication techniques.

## Introduction

The perioperative environment is complex and may be challenging for patients and guardians to navigate. The emotional burden and stressors inherent to the perioperative process commonly result in preoperative anxiety. Up to 75% of children and 50%‐75% of guardians experience preoperative anxiety [1,2]. Indeed, a mutual influence between the patient-guardian dyad can exacerbate or ameliorate the anxiety [3]. Preoperative anxiety is not benign; it is associated with difficult induction, an increased requirement for induction agents, intraoperative hemodynamic instability, emergence delirium, poorer pain control, prolonged recovery, and a greater risk of postoperative complications [4,5]. Additionally, preoperative anxiety may lead to behavioral changes that persist beyond the postoperative period, with up to 88% of children experiencing sleep and eating disturbances, enuresis, temper tantrums, or separation anxiety [6,7]. Though these maladaptive behaviors are most common in the first 2 weeks following anesthesia, they may persist for up to a year [7-9].

Educating the child and guardian about anesthesia, surgery, and postoperative recovery has been shown to improve preoperative anxiety [10]. Traditionally, preoperative education is provided to the guardian in written form in the surgery clinic, by a nurse phone call in the days leading up to surgery, and on the day of the procedure by preoperative nurses and anesthesiologists. However, production pressures, limited time, conflicting or inaccurate internet-based resources, and poor health literacy impede optimal coaching and effective communication with patients and their guardians in these settings [11-13]. Furthermore, despite best efforts to provide preoperative education, most children experience significant fear of the unfamiliar environment in the operating room.

Virtual reality (VR) presents a unique opportunity to support pediatric patients during the perioperative period by providing an immersive and engaging way to familiarize them with the often-intimidating process of anesthesia induction. Previous studies have demonstrated the effectiveness of various VR technologies in reducing pain and anxiety among children in stressful clinical settings [14-18]. Building on this foundation, our study aimed to evaluate the impact of a VR-based educational tool on preoperative anxiety in both children and their guardians. We hypothesized that exposure to the VR experience would result in reduced anxiety levels for children and guardians prior to surgery.

## Methods

### Overview

Following institutional review board ethical approval, we performed a single-center prospective randomized controlled trial including children 6‐12 years of age, presenting for ambulatory tonsillectomy and/or adenoidectomy, with or without bilateral ear tube insertion. We followed CONSORT (Consolidated Standards of Reporting Trials) guidelines for reporting randomized controlled trials and registered the study on ClinicalTrials.gov (NCT05008107). We consented and enrolled patients from October 2021 to September 2023. Patients were excluded if they were scheduled for inpatient admission, had undergone a procedure at our facility within the previous year (to limit the influence of prior familiarity with the perioperative process), were scheduled to undergo additional procedures alongside their primary surgery, or had been prescribed premedication for anxiolysis. VR interventions may pose challenges for individuals with sensory sensitivities, posttraumatic stress disorder, or other neurocognitive conditions due to the immersive and potentially overstimulating nature of the technology. To mitigate these risks, patients with a history of severe motion sickness, visual impairment, or known sensory processing disorders were excluded from participation. Additionally, preprocedural screening included a brief clinical interview to assess conditions such as posttraumatic stress disorder or anxiety disorders that could be exacerbated by immersive experiences. Given that the VR instructional tool was only available in English, we limited our study to children and guardians using English as their language of care.

The patient and their guardian arrived to the hospital approximately 1 hour prior to the scheduled procedure. Following registration, a preoperative nurse confirmed medications and allergies, took vital signs, and brought the patient and guardians to the preoperative holding area. Enrollment was done approximately 30‐45 minutes before the procedure, and informed consent was obtained from the patient’s guardian by a member of the research team. Patients were then block randomized to either receive VR exposure therapy or serve as controls (no intervention). Research team members were not blinded to group allocation. The primary outcome was patient anxiety. Baseline anxiety was assessed using the 6-item State-Trait Anxiety Inventory (6-STAI), a validated tool in pediatric populations [19]. Both patients and their guardians completed the 6-STAI on tablet devices provided by the research team (Table S1 in Multimedia Appendix 1) prior to the intervention. Research staff were not privy to individual responses. Patients assigned to the VR group viewed a proprietary instructional 5-minute video [20] using an Oculus (Meta) headset in the preoperative holding area. Guardians then viewed the same video. Following the VR exposure, the 6-STAI was readministered to both patients and guardians in the VR group to assess postintervention anxiety. The control group did not complete a second anxiety assessment, as only a brief period had elapsed between initial and potential follow-up measurements, making meaningful change unlikely. Following VR exposure (or in the absence of VR for the control group), the anesthesiologist and surgeon met with the patient and guardian to discuss the anesthetic plan and procedure. These discussions were not standardized. An OR nurse retrieved the patient just prior to surgery and separation from the guardian occurred just outside the doors to the operating room.

### VR Exposure

The VR program is a 360° video (non–computer generated) of a generic surgical center that provides an immersive first-person experience of the perioperative process. The video is narrated by a child and begins in the waiting room, welcoming the patient for their procedure. The patient can explore the waiting room and reception area as the narrator highlights the nil per os requirements that the patient should have abided by. The patient then enters a consultation room to meet with a pediatric anesthesiologist. The narrator explains the role of the pediatric anesthesiologist. The patient then takes a ride on a gurney through the operating room halls to the operating room. The patient can explore the room as the anesthesiologist introduces various tools used to provide anesthesia, including the face mask. The patient then lays back on the operating room table and the narrator introduces the blood pressure cuff, pulse oximeter, and electrocardiogram leads. The anesthesiologist places the mask over the patient’s face to induce anesthesia. Finally, the patient finds themselves in the recovery room waking up with a nurse and anesthesiologist.

### Statistical Analysis

We conducted statistical analysis to compare demographic characteristics and outcome measures between the VR and non-VR groups. Descriptive statistics were calculated for demographic characteristics within each group. The Shapiro-Wilk test was used to assess the assumption of normality for continuous variables. Since continuous variables did not adhere to a normal distribution, they were presented as median and interquartile ranges. Group comparisons for continuous variables were made using the Mann-Whitney *U* test. Categorical variables were summarized as counts and percentages, and appropriate statistical tests, such as the *Χ*^2^ test or Fisher exact test, were used for group comparisons. An ordinal logistic regression analysis was conducted to compare each of the 6-STAI component measures between the VR and non-VR groups, after testing for the proportionality odds assumption. This involved estimating odds ratios (OR) and 95% CIs to assess the association between the VR exposure and preoperative anxiety in pediatrics. Additionally, based on the validated correlation between the 6-STAI and the full 20-item version—as established by Marteau and Bekker [21]—we calculated a prorated STAI score by multiplying the 6-STAI score by a factor of 20/6. This yielded an estimated full-scale STAI score ranging from 20 to 80 [21]. We then categorized the prorated STAI scores into three groups: low anxiety (20-37), moderate anxiety (38-44), and high anxiety (45-80). Finally, we performed an ordinal logistic regression of the prorated STAI scores to assess the association between the VR exposure and preoperative anxiety. All statistical analyses were performed using Stata (version 18; StataCorp). A *P* value <.05 was adopted to determine statistical significance in all analyses.

A post hoc power analysis was conducted using the prorated STAI scores (20‐80 range). With 51 participants in the VR group and 56 in the control group, the study had approximately 82% power to detect a clinically meaningful difference of 5 points in anxiety scores. This calculation assumed a standard deviation of 8.9 (based on a previous study by Rizzo et al [22]), a 2-sided α level of .05, and a 2-sample comparison.

### Ethical Considerations

The study was approved by the ethical committee at Nationwide Children’s Hospital. Patients’ verbal assent and legal guardians’ signed consent were obtained. No compensation was provided to participants.

## Results

### Study Population Characteristics

**Figure 1.**
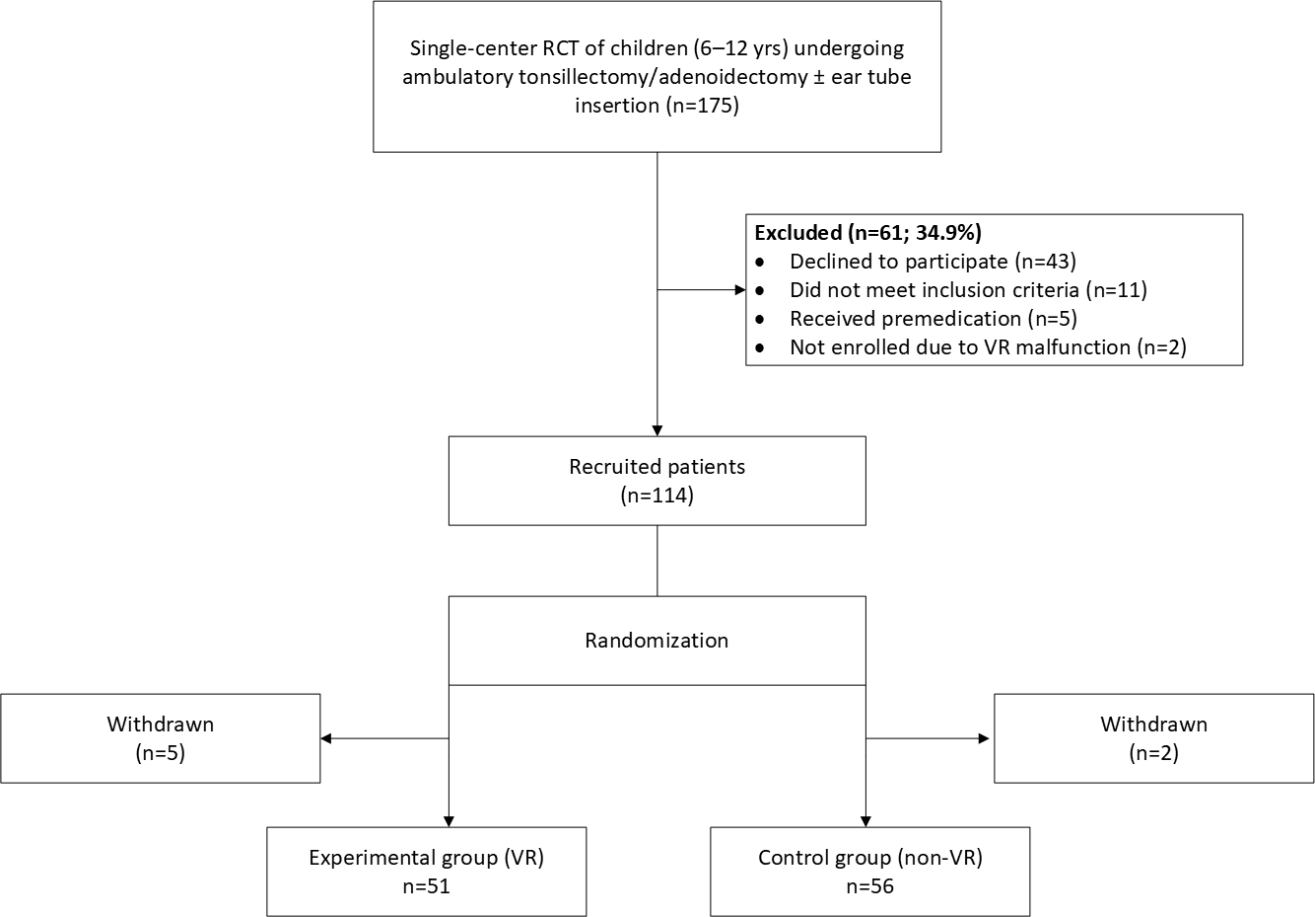
Patient selection flowchart. RCT: randomized controlled trial; VR: virtual reality.

Of the 175 children assessed for eligibility, 107 patients met the inclusion criteria and consented to participate (Figure 1).

Fifty-one children were randomized to the VR group and 56 to the control group. The average age of children in the study was 8 years, with no significant difference observed between the VR and non-VR groups. The majority of children were female (n=60, 56%) and non-Hispanic White (n=79, 76%). Baseline characteristics between the study and control groups were comparable (Table 1).

**Table 1.** Participant demographic data (N=107).

Characteristics	Entire cohort	Non-VR^a^	VR	*P* value^b^
Study population, n (%)	107 (100)	56 (52.3)	51 (47.7)	
Age (years), median (IQR)	8 (7-9)	8 (7-10)	8 (7-9)	.91
Gender, n (%)				.82
Female	60 (56.1)	32 (57.1)	28 (54.9)	
Male	47 (43.9)	24 (42.9)	23 (45.1)	
Race and ethnicity, n (%)				.44
Hispanic	4 (3.7)	3 (5.4)	1 (2)	
Non-Hispanic Black	17 (15.9)	8 (14.3)	9 (17.6)	
Non-Hispanic White	79 (73.8)	43 (76.8)	36 (70.6)	
Other/unknown	7 (6.5)	2 (3.6)	5 (9.8)	
Weight (kg), median (IQR)	31.3 (24.6-42.0)	32.3 (26.5-43.7)	30.9 (23.7-38.2)	.34
Height (cm), median (IQR)	131.1 (125.3-139.3)	131.1 (125.4-141.6)	131.8 (124-139)	.78
BMI (kg/m^2^), median (IQR)	18.0 (15.6-22.8)	18.8 (15.5-23.5)	17.2 (15.8-20.8)	.18

a VR: virtual reality.

b Group comparison was made using the *Χ*2 test or Fisher exact test for categorical variables and Mann-Whitney *U* test for continuous variables.

### Baseline (Preintervention) Anxiety

Patients in the VR and non-VR groups had similar preintervention anxiety scores across most STAI components, including feeling calm (*P*=.57), tense (*P*=.28), relaxed (*P*=.22), content (*P*=.27), and worried (*P*=.14). However, children in the non-VR group were significantly more likely to feel upset at baseline than those in the VR group (*P*=.01; Table S1 in Multimedia Appendix 1).

There was no difference between guardian anxiety scores at baseline between the VR and non-VR groups (Table S2 in Multimedia Appendix 1).

### Effect of VR on Anxiety by 6-STAI Component

**Figure 2.**
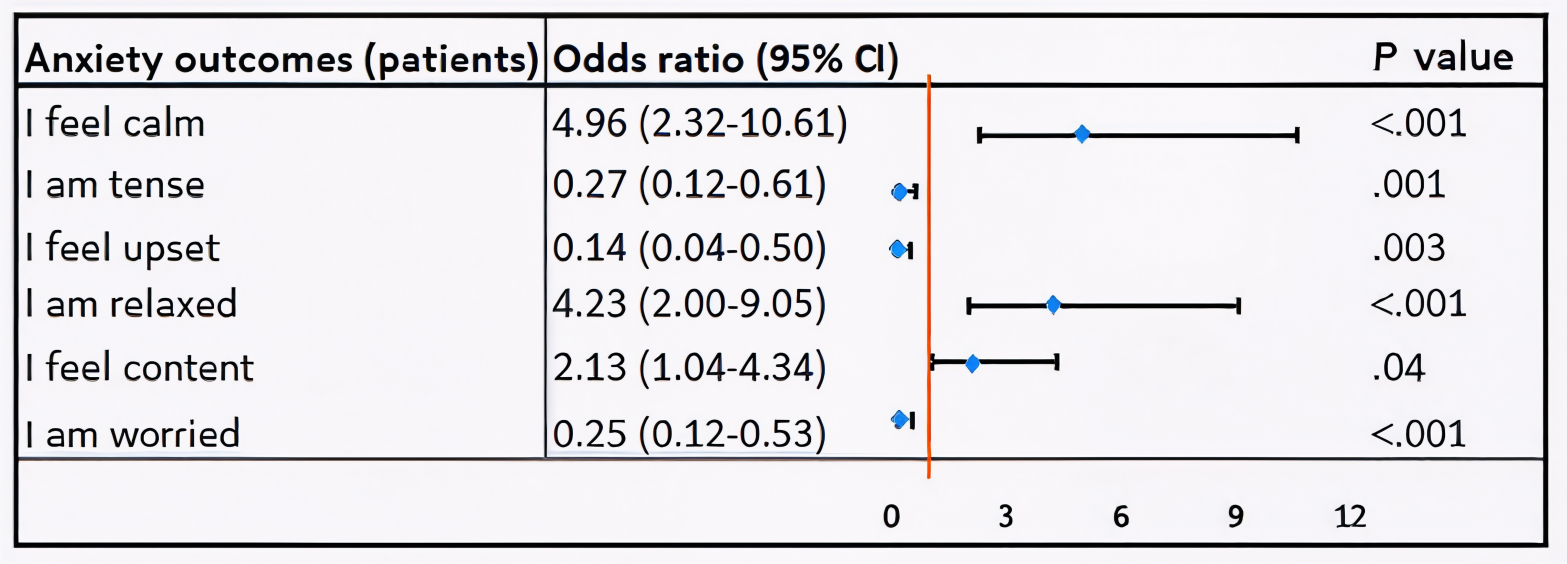
Ordinal logistic regression analysis examining the impact of virtual reality on patient preoperative anxiety.

**Figure 3.**
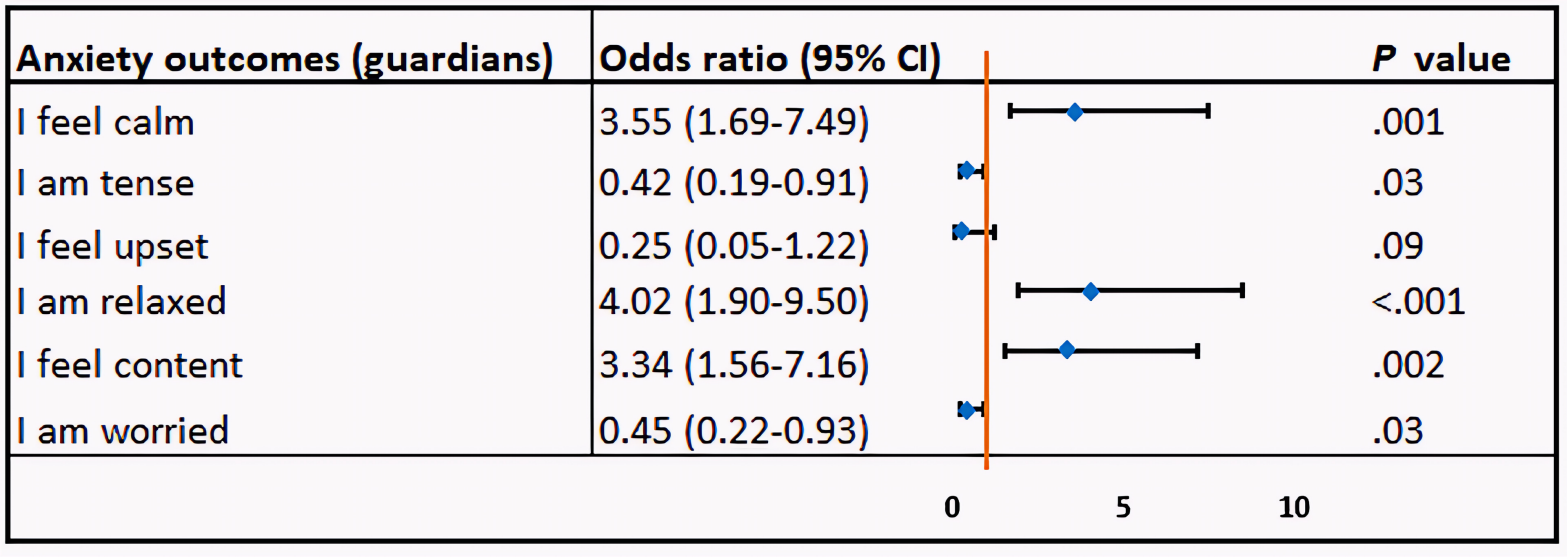
Ordinal logistic regression analysis examining the impact of virtual reality on guardian preoperative anxiety.

The results of the ordinal logistic regression analysis (Figure 2) revealed that, for patients, the estimated cumulative odds of feeling very calm (OR 4.95, 95% CI 2.32‐10.61; *P*<.001), relaxed (OR 4.23, 95% CI 2.00‐9.05; *P*<.001), and content (OR 2.13, 95% CI 1.04‐4.34; *P*=.04) were higher for those who used VR compared to those who did not. Conversely, the estimated cumulative odds of feeling tense (OR 0.27, 95% CI 0.12‐0.61; *P*=.001), upset (OR 0.14, 95% CI 0.04‐0.50; *P*=.003), and worried (OR 0.25, 95% CI 0.12‐0.53; *P*<.001) were significantly lower in patients who used VR. Similarly, for guardians, those who received VR reported higher odds of feeling very calm (OR 3.55, 95% CI 1.69‐7.49; *P*=.001), relaxed (OR 4.02, 95% CI 1.90‐8.50; *P*<.001), and content (OR 3.34, 95% CI 1.56‐7.16; *P*=.002) compared to those who did not receive VR. Additionally, they had lower odds of feeling tense (OR 0.42, 95% CI 0.19‐0.91; *P*=.03) and worried (OR 0.45, 95% CI 0.22‐0.93; *P*=.03). However, no statistically significant difference was found for feeling less upset (OR 0.25, 95% CI 0.05‐1.22; *P*=.09) between guardians who received VR and those who did not (Figure 3).

### Effect of VR on Prorated STAI Anxiety Level

The median prorated STAI scores were higher in the control group for both patients (37) and their guardians (40) compared those who received VR (patients: 33; guardians: 33). Among the VR group, median anxiety scores decreased following exposure: for patients, from 33 (pre-VR) to 30 (post-VR), and for guardians, from 33 to 27. VR exposure was also associated with a reduced proportion of patients with high anxiety from 25.5% to 9.8% and moderate anxiety from 7.8% to 2%. Similarly, the proportion of guardians reporting high anxiety dropped from 32.7% to 6.4%, and moderate anxiety dropped from 12.2% to 8.5% (Tables S3 and S4 in Multimedia Appendix 1).

### Effect of VR on the Odds of a High or Moderate Anxiety Level

The additional analysis of the prorated STAI score showed that after the intervention, patients exposed to VR had 85% lower odds of having high or moderate preoperative anxiety than those in the control group (OR 0.15, 95% CI 0.05‐0.41; *P*<.001). A similar pattern was observed in guardians, where those exposed to VR had significantly lower odds of high or moderate anxiety relative to those not exposed to VR (OR 0.14, 95% CI 0.06‐0.38; *P*<.001).

## Discussion

In this prospective randomized controlled trial, we demonstrated the ability of VR-based preoperative instruction to decrease anxiety of children undergoing ambulatory tonsillectomy. Specifically, the patients and guardians exposed to the VR tool were significantly less likely to experience moderate or high preoperative anxiety than those in the control group. They were also more likely to report feeling calm, relaxed, and content prior to surgery than those who were not exposed to VR and were less likely to report feeling tense, upset, and worried. These findings may represent an advance in pediatric perioperative care—preoperative anxiety remains a hurdle for pediatric anesthesiologists and their patients, with the optimal panacea yet to be identified. Many anesthesiologists rely on pharmacologic interventions to ease the child’s stress. VR technology may offer an effective alternative to pharmacologic premedication, without the risk of adverse effects.

Much of the existing pediatric VR research centers on distraction-based techniques, which aim to divert attention away from stressful stimuli using immersive and engaging VR experiences [23-26]. This approach has shown effectiveness in various clinical settings, including peripheral intravenous placement, anesthesia induction, and preoperative preparation [17,27,28]. In contrast, our study used VR-based exposure therapy, which uses virtual environments to simulate a patient’s specific stressor—in this case, the perioperative setting—to promote familiarity and reduce anxiety [29]. Research on VR exposure therapy for preoperative anxiety remains limited. For example, Eijlers et al [30] found no significant reduction in preoperative anxiety for children or guardians using VR exposure therapy compared to those who did not use VR; however, children in the VR group required fewer rescue analgesics postoperatively compared to controls. Conversely, Ryu et al [31] reported significantly lower preoperative anxiety and improved anesthesia induction compliance in children who received VR exposure therapy compared to those in the control group.

Anxiety reduction following our VR exposure intervention can likely be attributed to both desensitization—reducing anticipatory fear and distress—and information delivery [32]. Studies have shown that educational information improves both anxiety and guardian satisfaction [30,33,34]. Furthermore, data suggest that children desire more information about the perioperative experience, including details of the surgical environment, presence of their guardian, conduct of anesthesia, and expectations for postoperative pain. VR exposure therapy can provide the desired information in a fun and interactive platform [35].

Implementing VR in the perioperative environment is both feasible and potentially cost-effective, particularly when thoughtfully integrated into existing clinical workflows. VR interventions can be administered by either nurses or child life specialists, depending on institutional resources. Both roles are well-suited to deliver the experience with minimal additional training. The intervention itself is brief—typically requiring only about 5 minutes—making it easy to incorporate into standard preoperative routines. From an infrastructure perspective, the requirements are modest—ideally, there should be one VR headset per operating room to ensure consistent access for all patients. Most commercial VR headsets cost under US $500, making them a realistic investment for hospitals. Maintenance is straightforward, with simple hygiene protocols such as disinfecting the headset between uses with alcohol swabs. Moreover, the flexibility of the intervention allows it to be implemented not only on the day of the surgery but also in preoperative clinics or during earlier surgical consultations. This expanded access provides families with multiple opportunities to familiarize themselves with the perioperative process, supporting anxiety reduction while maintaining clinical efficiency.

It is important to note the limitations of our study. First, although an a priori sample size calculation was not performed, a post hoc power analysis confirmed that the study was adequately powered to detect a clinically meaningful difference in preoperative anxiety between groups. Our study was limited to school-aged children undergoing tonsillectomy and/or adenoidectomy. However, preoperative anxiety afflicts children of all ages and varies with type of surgery, anesthetic, and expected disposition [4]. Therefore, our findings may not be generalizable to all populations of children undergoing surgery. Additionally, the STAI questionnaire, which is the most frequently used anxiety assessment, is quite extensive and may lead to response bias [4]. Postintervention anxiety was only assessed in the VR group, introducing potential confounding. Anxiety is dynamic; therefore, it is conceivable that STAI scores could fluctuate after elapsed time or following additional interactions with health care providers prior to the surgery. Although virtual reality exposure therapy appears to be an effective tool for reducing pediatric preoperative anxiety, it has yet to be directly compared to the current gold standard for anxiolysis: midazolam. Future studies should assess the comparative effectiveness of pharmacologic interventions versus VR-based approaches in reducing preoperative anxiety. Additionally, the potential impact of VR exposure on postoperative pain and analgesic requirements should be explored. To enhance accessibility and equity, VR tools should be developed for patients who receive care in languages other than English. With these considerations in mind, the findings of this study suggest that VR exposure therapy may be a valuable intervention for reducing anxiety in both pediatric patients and their guardians.

In conclusion, this single-center randomized controlled trial demonstrated that VR exposure therapy may have a beneficial effect on reducing preoperative anxiety in both pediatric patients and their guardians. By providing an immersive, nonpharmacologic, and engaging preparatory experience, VR may serve as a valuable adjunct to traditional perioperative care. These findings suggest that VR has the potential to enhance the surgical experience by addressing emotional well-being in a child- and family-centered manner. Although further research is needed, this study contributes to a growing body of evidence supporting the role of digital therapeutics in pediatric perioperative care.

## Supplementary material

10.2196/73392 Multimedia Appendix 1 Supplemental tables showing preintervention and postintervention anxiety in patients and guardians as assessed by the 6-State-Trait Anxiety Inventory

10.2196/73392 Checklist 1 CONSORT checklist.

## Abbreviations

CONSORT: Consolidated Standards of Reporting Trials
OR: odds ratio
STAI-6: 6-item State-Trait Anxiety Inventory
VR: virtual reality
