# Supplementary material for: Virtual Reality Exposure Therapy and Patient Education for Preoperative Anxiety in Pediatrics: Randomized Controlled Trial
Source: JMIR Perioper Med. 2025 Oct 27;8:e73392. doi: 10.2196/73392 (PMC12558565; doi:10.2196/73392)
Supplement: Multimedia Appendix 1 [file periop-v8-e73392-s001.docx]

**Supplemental Table 1. Pre-intervention (Pre-VR) patient anxiety as assessed by the 6-State-Trait Anxiety Inventory**

| **Characteristics** | | **Non-VR** | **VR** | **P-value^a^** |
| --- | --- | --- | --- | --- |
| **Study population** | | 56 (52.3%) | 51 (47.7%) |  |
| **I feel calm, n (%)** | |  |  | 0.57 |
|  | Very much | 14 (25.0) | 15 (29.4) |  |
|  | Moderately so | 18 (32.1) | 15 (29.4) |  |
|  | Somewhat | 14 (25.0) | 15 (29.4) |  |
|  | Not at all | 10 (17.9) | 6 (11.8) |  |
| **I am tense, n (%)** | |  |  | 0.28 |
|  | Very much | 6 (10.7) | 6 (11.8) |  |
|  | Moderately so | 6 (10.7) | 7 (13.7) |  |
|  | Somewhat | 23 (41.1) | 10 (19.6) |  |
|  | Not at all | 21 (37.5) | 28 (54.9) |  |
| **I feel upset, n (%)** | |  |  | 0.01 |
|  | Very much | 3 (5.4) | 1 (2.0) |  |
|  | Moderately so | 4 (7.1) | 3 (5.9) |  |
|  | Somewhat | 11 (19.6) | 1 (2.0) |  |
|  | Not at all | 38 (67.9) | 46 (90.2) |  |
| **I am relaxed, n (%)** | |  |  | 0.22 |
|  | Very much | 18 (32.1) | 23 (45.1) |  |
|  | Moderately so | 17 (30.4) | 13 (25.5) |  |
|  | Somewhat | 12 (21.4) | 8 (15.7) |  |
|  | Not at all | 9 (16.1) | 7 (13.7) |  |
| **I feel content, n (%)** | |  |  | 0.27 |
|  | Very much | 22 (39.3) | 24 (47.1) |  |
|  | Moderately so | 12 (21.4) | 10 (19.6) |  |
|  | Somewhat | 10 (17.9) | 12 (23.5) |  |
|  | Not at all | 12 (21.4) | 5 (9.8) |  |
| **I am worried, n (%)** | |  |  | 0.15 |
|  | Very much | 16 (28.6) | 8 (15.7) |  |
|  | Moderately so | 7 (12.5) | 5 (9.8) |  |
|  | Somewhat | 15 (26.8) | 18 (35.3) |  |
|  | Not at all | 18 (32.1) | 20 (39.2) |  |
| ^a^ P-values were obtained from ordinal logistic regression. | | | | |

**Supplemental Table 2. Pre-intervention (Pre-VR) guardian anxiety as assessed by the 6-State-Trait Anxiety Inventory**

| **Characteristics** | | **Non-VR** | **VR** | **P-value^a^** |
| --- | --- | --- | --- | --- |
| **Study population** | | 55 (51.4) | 52 (48.6) |  |
| **I feel calm, n (%)** | |  |  | 0.50 |
|  | Very much | 16 (29.1) | 16 (31.4) |  |
|  | Moderately so | 17 (30.9) | 18 (35.3) |  |
|  | Somewhat | 19 (34.6) | 16 (31.4) |  |
|  | Not at all | 3 (5.4) | 1 (2.0) |  |
| **I am tense, n (%)** | |  |  | 0.58 |
|  | Very much | 1 (1.8) | 1 (1.9) |  |
|  | Moderately so | 10 (18.2) | 9 (17.3) |  |
|  | Somewhat | 17 (30.9) | 13 (25.0) |  |
|  | Not at all | 27 (49.1) | 29 (55.8) |  |
| **I feel upset, n (%)** | |  |  | 0.43 |
|  | Very much | 0 | 0 |  |
|  | Moderately so | 4 (7.4) | 3 (5.8) |  |
|  | Somewhat | 4 (7.4) | 2 (3.8) |  |
|  | Not at all | 46 (85.2) | 47 (90.4) |  |
| **I am relaxed, n (%)** | |  |  | 0.12 |
|  | Very much | 11 (20.4) | 16 (30.8) |  |
|  | Moderately so | 14 (25.9) | 15 (28.9) |  |
|  | Somewhat | 25 (46.3) | 19 (36.5) |  |
|  | Not at all | 4 (7.4) | 2 (3.8) |  |
| **I feel content, n (%)** | |  |  | 0.43 |
|  | Very much | 13 (24.5) | 15 (30.0) |  |
|  | Moderately so | 21 (39.6) | 20 (40.0) |  |
|  | Somewhat | 18 (34.0) | 15 (30.0) |  |
|  | Not at all | 1 (1.9) | 0 |  |
| **I am worried, n (%)** | |  |  | 0.68 |
|  | Very much | 2 (3.6) | 3 (5.8) |  |
|  | Moderately so | 16 (29.1) | 12 (23.1) |  |
|  | Somewhat | 17 (30.9) | 16 (30.8) |  |
|  | Not at all | 20 (36.4) | 21 (40.4) |  |
| ^a^ P-values were obtained from ordinal logistic regression. | | | | |

**Supplemental Table 3. Pre-intervention (Pre-VR) Anxiety Level, Patient and Guardian**

| **Prorated STAI Anxiety Level** | | **Non-VR** | **VR** |
| --- | --- | --- | --- |
| **Patient** | |  |  |
|  | Low | 29 (51.8) | 34 (66.7) |
|  | Moderate | 7 (12.5) | 4 (7.8) |
|  | High | 20 (35.7) | 13 (25.5) |
| **Guardian^a^** | |  |  |
|  | Low | 24 (46.2) | 27 (55.1) |
|  | Moderate | 9 (17.3) | 6 (12.2) |
|  | High | 19 (36.5) | 16 (32.7) |
| Counts (percentages) of those experiencing low (STAI score 20-37), moderate (STAI score 38-44), and high (STAI score 45-80) levels of anxiety prior to the VR intervention in both groups.  ^a^ Differences in “n” are due to missing item responses; participants with any missing item lacked a total score. | | | |

**Supplemental Table 4. Post-intervention (Post-VR) Anxiety Level, Patient and Guardian**

| **Prorated STAI Anxiety Level** | | **Non-VR^b^** | **VR** |
| --- | --- | --- | --- |
| **Patient** | |  |  |
|  | Low | 29 (51.8) | 45 (88.2) |
|  | Moderate | 7 (12.5) | 1 (2.0) |
|  | High | 20 (35.7) | 5 (9.8) |
| **Guardian^a^** | |  |  |
|  | Low | 24 (46.2) | 40 (85.1) |
|  | Moderate | 9 (17.3) | 4 (8.5) |
|  | High | 19 (36.5) | 3 (6.4) |
| Counts (percentages) of those experiencing low (STAI score 20-37), moderate (STAI score 38-44), and high (STAI score 45-80) levels of anxiety following VR intervention in the VR-group.  ^a^ Differences in “n” are due to missing item responses; participants with any missing item lacked a total score.  ^b^ The non-VR group did not complete a second administration of the STAI; therefore, post-intervention scores are identical to pre-intervention values. | | | |
